# Promoting teachers' wellbeing through a serious game intervention: a qualitative exploration of teachers' experiences

**DOI:** 10.3389/fpsyg.2024.1339242

**Published:** 2024-03-27

**Authors:** Valeria Cavioni, Elisabetta Conte, Veronica Ornaghi

**Affiliations:** ^1^Department of Social Sciences, University of Foggia, Foggia, Italy; ^2^Department of Human Sciences for Education “Riccardo Massa”, University of Milano-Bicocca, Milan, Italy

**Keywords:** teachers' wellbeing, teacher training, serious game, social and emotional competencies, qualitative study, European project, Teaching to Be

## Abstract

**Introduction:**

Teachers' wellbeing plays a critical role in their overall job satisfaction, motivation, and effectiveness in building supporting learning environments. In today's dynamic educational settings, where teachers often face numerous challenges and stressors, their wellbeing becomes increasingly vital. Consequently, there is an urgent need to innovate and develop targeted training interventions that can support specifically the wellbeing of educators.

**Methods:**

This study sought to provide an overview of the “Online Wellbeing Course - OWC,” a serious game developed to enhance teachers' wellbeing, and to investigate the participants' feedback after being engaged in the OWC, utilizing a qualitative approach through focus group discussions. A total of 189 in-service teachers took part in the study. To qualitatively explore their experiences with the OWC, participants were involved in focus groups and asked to provide feedback about how and to what extent the course was beneficial for their wellbeing.

**Results:**

Teachers reported enhancements in areas such as emotional competence, self-care strategies, social awareness, relationship skills, decision-making, and school climate.

**Discussion:**

These outcomes suggested the potential of serious games as an innovative training approach for supporting teachers' wellbeing, offering valuable insights for researchers, policymakers, and educators.

## 1 Introduction

Teachers' wellbeing has been articulated as a complex and evolving construct that is deeply rooted in the interplay of individual, familial, and communal beliefs, values, and experiences. As delineated by McCallum et al. (2017), this comprehensive definition asserts that wellbeing spans across physical, psychological, and emotional domains, playing a key role in the welfare of both educators and their students within the educational settings. Teacher professional wellbeing is characterized by the equilibrium between work and leisure activities that foster health and personal fulfillment, which is influenced by a multitude of factors, including personal teacher capabilities, social and emotional competencies, personal responses to workload and work conditions, and professional relationships (Nwoko et al., 2023). Personal capabilities encompass resilience, self-efficacy, independence, and coping mechanisms (Chaudhuri et al., 2021; Sohail et al., 2023) alongside social and emotional skills that involve recognizing and effectively managing one's own emotions as well as those of others (Skinner et al., 2021; Wang et al., 2022). Responses to workload and working conditions can comprise experiences of burnout, fatigue, exhaustion, stress, unattainable expectations, bureaucratic challenges, and feeling excluded from decision-making processes (Carroll et al., 2022; Ornaghi et al., 2023). Additionally, relational elements involve challenges such as student misconduct, conflicts with parents and colleagues, and a perceived absence of support from the school administration (Aldrup et al., 2018; Conte et al., 2024).

Numerous studies have shown that the teaching profession is among those with the highest levels of work-related stress since teachers are often exposed to a series of difficulties and challenging situations that can negatively affect their mental wellbeing (Chang, 2009; OECD, 2019). The workload, the management of students' social, emotional, and behavioral problems in the classroom, the responsibility for their physical and psychological health, the partnership between the school and the family as well as the low salary are listed as the most significant stressors which require considerable emotional effort (Cavioni and Grazzani, 2023; Cavioni et al., 2023b; Conte et al., 2024). Furthermore, the COVID-19 pandemic has profoundly impacted societies worldwide, with widespread disruption and unprecedented challenges in various domains, including education (Colomeischi et al., 2022; Martinsone et al., 2022). Therefore, teachers, as frontline educators, had to face numerous adversities such as the sudden shift to remote learning models and adapting their teaching methods (Lee and Yin, 2021). The risk of contagion, inadequate work conditions, lack of support from school leaders, difficult collaborations with parents, the absence of face-to-face interactions, and the loss of physical classroom settings were some of the most stressful factors they perceived (Pressley, 2021; Collie, 2022; Messineo and Tosto, 2022; Quinn et al., 2022). Consequentially, teachers experienced increased psychological issues heightening levels of stress, anxiety, and emotional exhaustion (Lizana and Vega-Fernandez, 2021; Lizana et al., 2021; Truzoli et al., 2021).

Research has found that supporting the development of teachers' social and emotional skills - such as the ability to recognize and regulate their own emotions (particularly anxiety and stress), collaboration, empathy, self-efficacy, and the ability to seek and maintain social support - increase their wellbeing (Pulido-Martos et al., 2016; Collie et al., 2020; Mariani et al., 2020; Ornaghi et al., 2022, 2023). In turn, positive effects may be visible also on students' achievements, motivation, school belonging, and mental health (Harding et al., 2019; Berg et al., 2021; Cefai et al., 2022). While previous research has recognized the importance of focusing on teachers' wellbeing (Cavioni et al., 2020; Lester et al., 2020; Hascher et al., 2021), still limited attention has been given to the development and implementation of targeted interventions that directly address the unique challenges faced by educators (Cavioni et al., 2023b). Given this background, more research is needed to provide specific training and support to teachers in developing such key skills to cope with daily job challenges.

To address this gap in the literature and respond to the pressing need for innovative interventions, this study aims to explore the potential of a serious game intervention in enhancing teachers' wellbeing. Accordingly, the objective of this study is twofold: (1) to describe the serious game developed within the “Teaching to Be” European project (https://teachingtobe.eu/), namely the “Online Wellbeing Course – OWC,” aimed at sustaining teachers' wellbeing; (2) to explore Italian teachers' experiences with the OWC, applying a qualitative approach, specifically through focus group discussions.

## 2 Educational potential of serious games

Serious games consist of educational video games that involve and guide the player to achieve predefined objectives (Buendía-García et al., 2013). More specifically, “*serious games are interactive digital activities which, through virtual simulation, allow participants to have precise and accurate (even complex) experiences, capable of promoting active, participatory and engaging learning paths through the form of a game in the various domains of human existence*” (Anolli and Mantovani, 2011, p. 156). The main goal of serious games is to involve the players in solving problematic situations and guide the user in reflecting on their own experience and the contents of the game. Serious games have been often used to increase explicit knowledge and procedural skills related to professional competence (Thangavelu et al., 2022); these may also enhance psychological skills such as reasoning, problem-solving, ability to manage emotions, communication, and teamwork competencies (Westera et al., 2008).

Serious games are increasingly recognized for their educational potential, primarily due to their distinct characteristics of simulation, interactivity, and gamification (Gentry et al., 2019; Riva and Gaggioli, 2019). Indeed, serious games are intended to reproduce real situations and phenomena inside virtual environments to help players reflect on how to cope with present and future problems (Pallavicini et al., 2022a). This feature allows gamers to make mistakes without feeling the negative emotions that are usually experienced in problem-solving situations such as the fear, anxiety, and frustration of making errors (Beard and Wilson, 2006; Dalgarno and Lee, 2010). Simulation is, also, characterized by a high transferability and generalizability of acquired and practical knowledge and skills from the virtual to the real offline world since the scenarios proposed in serious games can offer a wide range of alternatives in response to a problematic situation, thus soliciting the user in the search for the optimal solution (Buendía-García et al., 2013). Interactivity is allowed by creating a dynamic and responsive environment that encourages users to actively interact with the contents by making choices and providing answers, rather than passive observers, promoting also a sense of agency and experiencing the consequences of their decisions (Salen and Zimmerman, 2004). Users are presented with complex scenarios and challenges that require them to develop attention, memory, problem-solving, critical thinking and the abilities to analyse information, make connections, and generate creative solutions (Van Der Spek et al., 2013; Lamb et al., 2018). Additionally, by incorporating elements such as storytelling, immersive environments, and interactive gameplay, serious games have the potential to evoke a wide range of emotions, namely joy, interest, surprise, and curiosity (Anolli et al., 2010; Shute et al., 2015a,b). These emotional experiences also encourage feelings of self-efficacy, commitment, and perseverance aimed at achieving the game's goals (Rooney, 2012; Wouters et al., 2013). Moreover, serious games are characterized by their gamification elements, where learning contents are delivered through interactive gameplay. This approach makes the learning process more engaging and rewarding (Seaborn and Fels, 2015; Sailer et al., 2017). Gamification enables users to track their progress in acquiring knowledge or skills and provides incentives through various game elements, such as rewards and scores, which are earned by successfully tackling challenges and advancing through different levels. What sets serious games apart is their deliberate incorporation of game-like features to immerse users in activities that traditionally may not be associated with gaming (Deterding, 2012). These features significantly enrich the educational experience by fostering heightened attention, enhanced problem-solving abilities, and intrinsic motivation (Csikszentmihalyi, 1990; Green et al., 2010; Procci et al., 2012; Liu, 2014).

Serious games have been widely used in different fields, including training and education of working staff in organizations to improve their learning and decision-making capacity, develop leadership skills and face challenges that are typical of specific sectors (e.g., Brandao et al., 2012; Tobler-Ammann et al., 2017; Kyaw et al., 2019). They have been also successfully utilized in healthcare simulations, where they provide medical professionals with opportunities to practice complex procedures and improve patient outcomes (Tudor Car et al., 2019). In the field of wellbeing promotion, they have been mainly employed by healthcare staff, especially following the COVID-19 pandemic, to manage and reduce stress, anxiety, and depression (van Gaalen et al., 2021; Pallavicini et al., 2022b).

In educational settings, serious games have predominantly been designed for student use, aiming to enhance their learning in various school subjects (Zhonggen, 2019; Cichy et al., 2020; Chen et al., 2023) or skills including the promotion of wellbeing and health-related topics (Arnab et al., 2013; Banos et al., 2013; Kolić-Vehovec et al., 2019). However, the development of serious games specifically designed for teachers' professional growth remains notably scarce. For instance, the “Kiddo” game is aimed at facilitating teacher-led discussions on gender equality in classrooms. It seeks to enlighten school-aged children about gender stereotypes by engaging them in diverse narrative-based scenarios that require decision-making (Yañez et al., 2023). The SRL-4Ts, another serious game for educators, draws inspiration from the Trivial Pursuit board game. Developed by Persico et al. (2023), this game is designed to encourage teachers to reflect on and develop collaborative activities aimed at enhancing students' self-regulated learning practices and skills. While these studies present serious games designed for use by teachers, their primary objectives are to enhance specific competencies in students rather than focusing directly on the professional development or personal skills of the teachers themselves. Indeed, to the best of our knowledge, serious games have not been used yet to specifically enhance teachers' professional wellbeing.

## 3 The “Online Wellbeing Course”

### 3.1 The development of the game

The development of the contents of the serious game took place in two phases. First, a detailed examination of the extant scholarly literature about teachers' wellbeing was conducted, with a specific focus on identifying at-risk and protective factors. This literature review served as a crucial foundation for informing the design and development of the game's contents, ensuring its relevance in promoting teachers' wellbeing. This involved examining studies that investigated the various factors influencing teachers' wellbeing considering both at risk-factors, including work-related stress, burnout, and emotional exhaustion, and protective factors, namely the core components of social and emotional learning (SEL) such as self-awareness, self-management, social awareness, relationship skills, and responsible decision-making (Mahoney et al., 2021; Ornaghi et al., 2023). The literature analysis also examined the importance of SEL for educators, its impact on teacher-student relationships, classroom climate, and students' wellbeing, as well as the potential consequences on the broader educational environment (Domitrovich et al., 2017; Cavioni et al., 2023a; Conte et al., 2023).

Next, a participatory action research (PAR) approach was employed to further improve the contents of the serious game (Genat, 2009). The PAR methodology focuses on anchoring research within the authentic experiences of individuals most impacted by the subject of study (Kemmis et al., 2014). Consequently, PAR strives to give power to its participants through their active involvement in the research process. A sample of teachers (*N* = 86; F = 77; mean age = 46.87 years; *SD* = 10.87, min = 19; max = 65) was engaged in a collaborative effort to revise and refine the game's contents. We utilized a convenience sampling method, a type of non-probability sampling technique. This approach was chosen as it allowed us to select participants from the general teacher community who were willing and available to participate (Emerson, 2015). The criteria for inclusion in the study were twofold: first, participants needed to be currently working as in-service teachers; second, they were required to agree to the terms of participation as outlined in the study.

The research team facilitated open and inclusive discussions to encourage the teachers' active engagement and contributions. The PAR consisted of a series of focus groups where the teachers, as co-researchers, were actively involved in providing feedback, suggestions, and insights on the game's contents. To facilitate focus group discussions, a semi-structured guide composed of a series of open-ended questions was developed (see Table 1). The questions were designed to not only gather feedback on the current state of the game but also to elicit constructive suggestions for improvements directly from the teachers, who were supposed to be the end-users of the game.

**Table 1:** Sample questions applied during the PAR phase.

**Questions**
• “Can you identify any elements within the game that might need enhancement to more effectively address your psychological needs? If so, how would you suggest improving them?”
• “Regarding the game's contents, which parts do you think could assist you in strengthening your relationships with colleagues, students, and parents? Additionally, do you have any suggestions for how these elements could be further developed or modified?”
• “Are there specific features of the game you think could be adapted into your teaching to boost student engagement and learning outcomes? Could you propose any ways to refine these aspects for classroom application?”

By actively involving this group of teachers via the PAR methodology, it ensured that the course's contents were not only relevant and practical but also resonated with the real-world challenges and scenarios faced by educators.

Two methods were employed to ensure comprehensive documentation of the discussions: audio recording and note-taking. Each focus group session was audio-recorded, with the explicit consent of all participating teachers. Simultaneously, written notes were taken by the researchers throughout the sessions. At the end of each session, participants were presented with a summary of the main conclusions for their verification, and to facilitate additional enhancements to the game's contents. The collection of teachers' feedback and suggestions played a crucial role in identifying areas that required further elaboration, clarifying concepts, and improving the overall user experience. This integration of research-based evidence and practitioner perspectives ensured that the game's contents were well-grounded, relevant, and responsive to the unique needs and challenges that teachers faced.

### 3.2 Contents of the OWC

The game's activities, scenarios, challenges, and interactive features were designed to facilitate engagement, reflection, skill development, and knowledge acquisition in the areas of teachers' wellbeing and SEL promotion. The game included 12 levels which were intended to be played over 5 months. Table 2 displays the OWC goals for each level.

**Table 2:** OWC objectives by level.

**Levels**	**Goals**
1	• To introduce the OWC • To understand why it is important to talk about wellbeing • To think about professional wellbeing • To reflect on the problems that teachers can and cannot solve
2	• To identify personal strengths and increase their use • To explain the S.M.A.R.T. methodology • To identify personal and professional goals • To monitor the steps to achieve goals
3	• To analyze the link between emotions, behaviors, and beliefs • To identify what can interfere with or hinder the development of quality social relationships at school
4	• To analyze the sources of social support in different life contexts • To understand the strategies that can be applied to create positive relational environments
5	• To change established habits for time management when they are ineffective • To put into practice new effective strategies for time management
6	• To reflect on the importance of using classroom management strategies • To identify effective strategies for classroom management • To implement new strategies for classroom management in daily practice
7	• To recognize stress responses • To accurately identify emotions • To explore stress management strategies
8	• To learn how to deal effectively with stressful situations • To effectively improve emotional responses to stressful situations • To learn how to get in touch with body sensations
9	• To develop self-empathy • To increase compassion and empathy for others • To value and respect others' points of view • To reflect on personal worth and appreciate diversity
10	• To develop communication skills • To be able to critically analyze, evaluate and reflect on one's professional situation and wellbeing
11	• To improve self-confidence • To develop leadership skills
12	• To reflect on work performance in order to achieve better results and increase productivity • To critically analyze challenging situations and find alternative constructive solutions

### 3.3 Features

The serious game included several features aimed at creating an immersive and engaging environment that facilitates self-reflection and learning about teachers' wellbeing and promotes active engagement, capturing, and maintaining teachers' attention throughout the gameplay. The OWC was designed for individual use; however, teachers were also encouraged to discuss and share their reflections and acquired knowledge with their colleagues, in offline school settings.

#### 3.3.1 Narrative and storytelling

The OWC had a narrative-type game structure in which each player had an avatar of a teacher to emotionally engage participants and create connections to their real-life experiences at school. The storytelling aspect stimulated self-reflection and teachers could empathize with their own and other characters. The serious game began with a short introductory video that intended to present the story and the setting to users.

#### 3.3.2 Immersive and interactive scenarios

**Figure 1:**
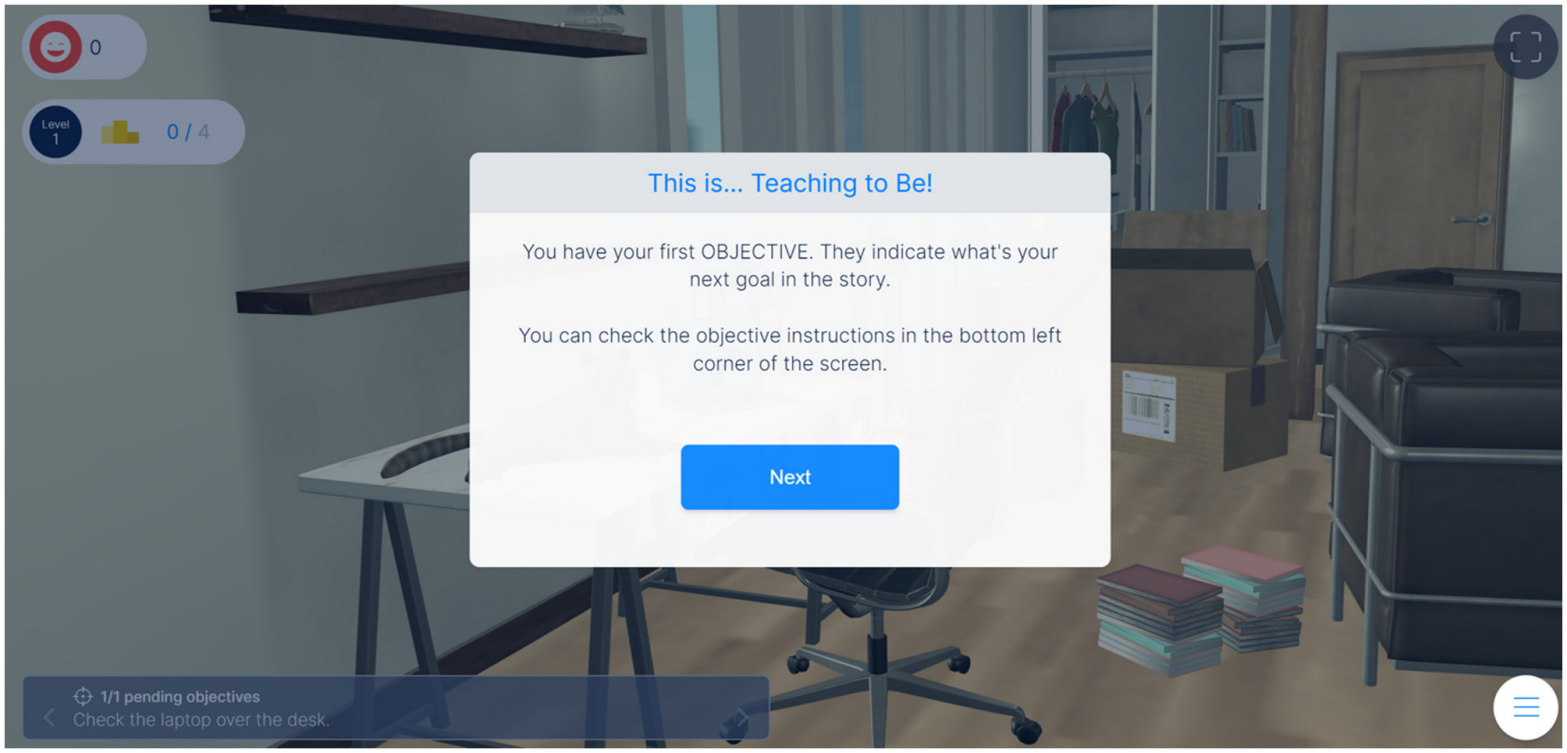
Instructions related to game objectives.

The game incorporated immersive scenarios that allowed teachers to step into various challenging situations related to their profession. The immersive nature of the game created a realistic and engaging environment for self-reflection and learning. By navigating these scenarios, teachers were prompted to reflect on their own wellbeing, get to know and practice coping strategies, consider different perspectives when facing difficult social problems, and reflect on the potential consequences of their actions in the school context. For instance, at the beginning of the game, the player was presented with instructions to reach the different game's scenarios and use the different tools, such as to get to know the specific objectives to be pursued (see Figure 1).

**Figure 2:**
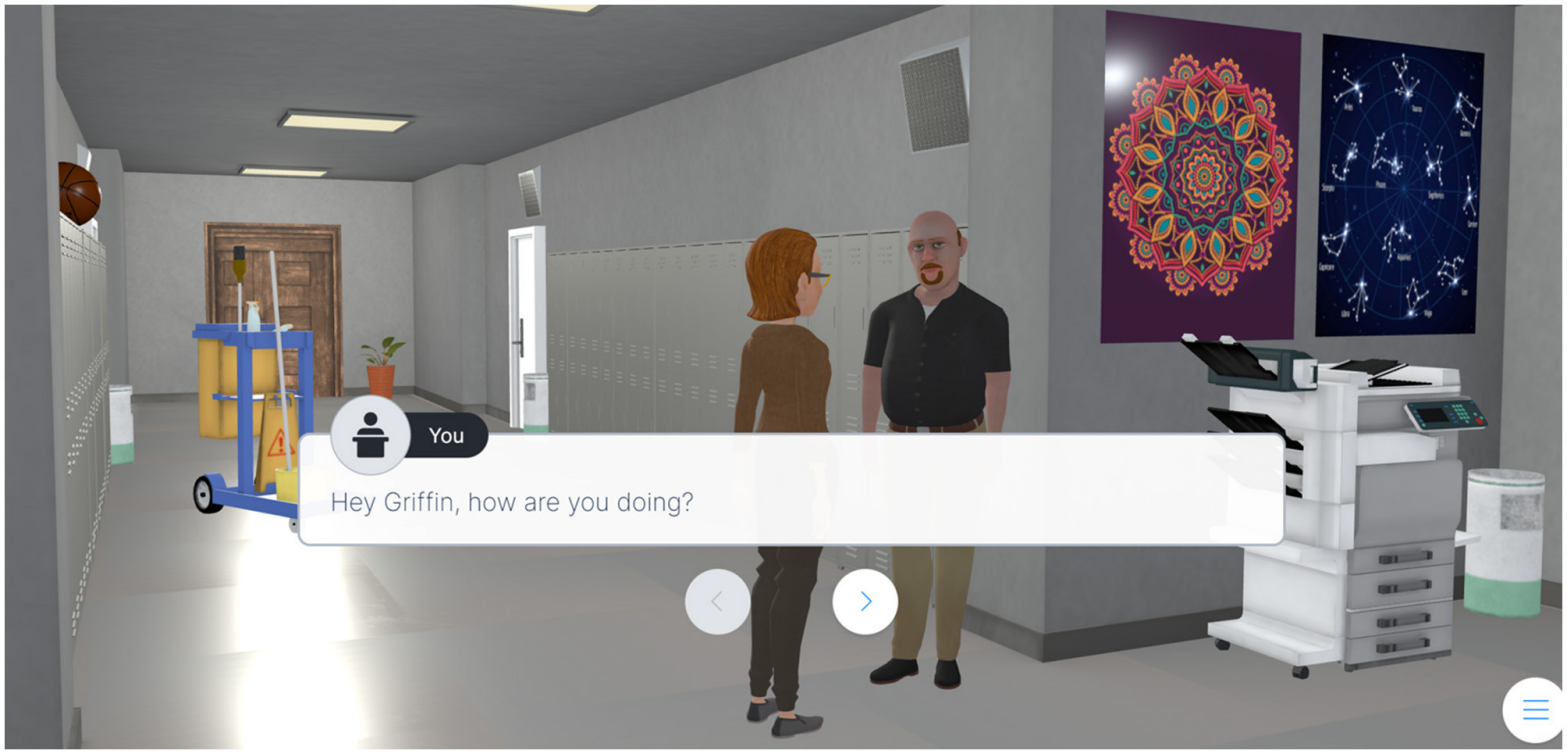
Example of interaction with a colleague.

Another notable feature of the game was the possibility for teachers to interact with various characters and objects within the virtual environment. These interactive elements were aimed to facilitate active exploration and provide immersive learning experiences that could be transferred to real-world scenarios. As a way of example, teachers could interact with virtual avatars representing colleagues or students to practice effective communication and empathy (see Figure 2).

**Figure 3:**
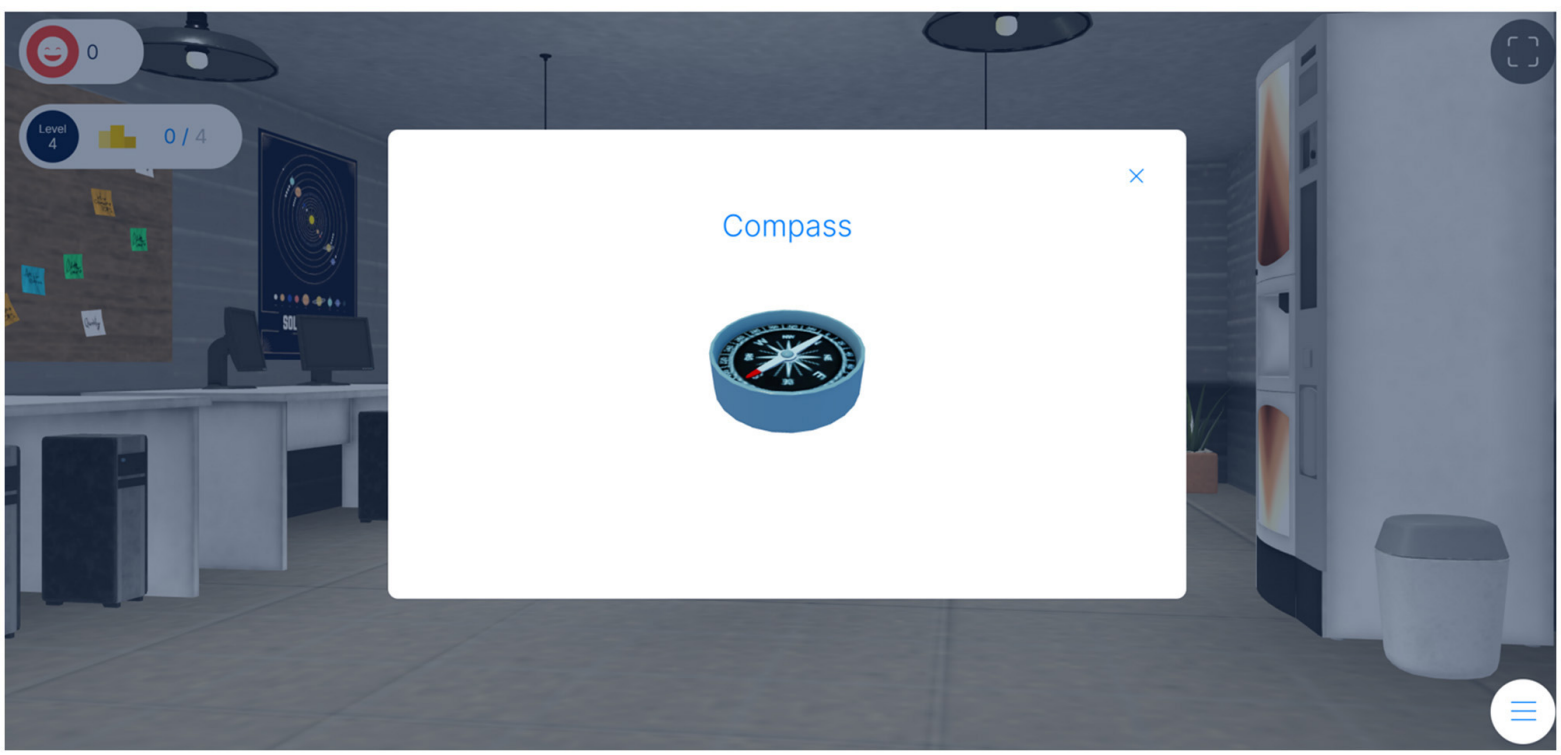
Example of an inventory item.

The OWC incorporated an inventory system that allowed teachers to collect and utilize various objects throughout their gameplay (e.g., a compass, Figure 3). Teachers could access their inventory at any time during gameplay to use the objects they had collected. These objects served as tools that teachers could use to overcome challenges and progress in the game. The inclusion of an inventory system encouraged teachers' problem-solving skills and creative thinking on the use of the inventory items.

#### 3.3.3 Reflection prompts

**Figure 4:**
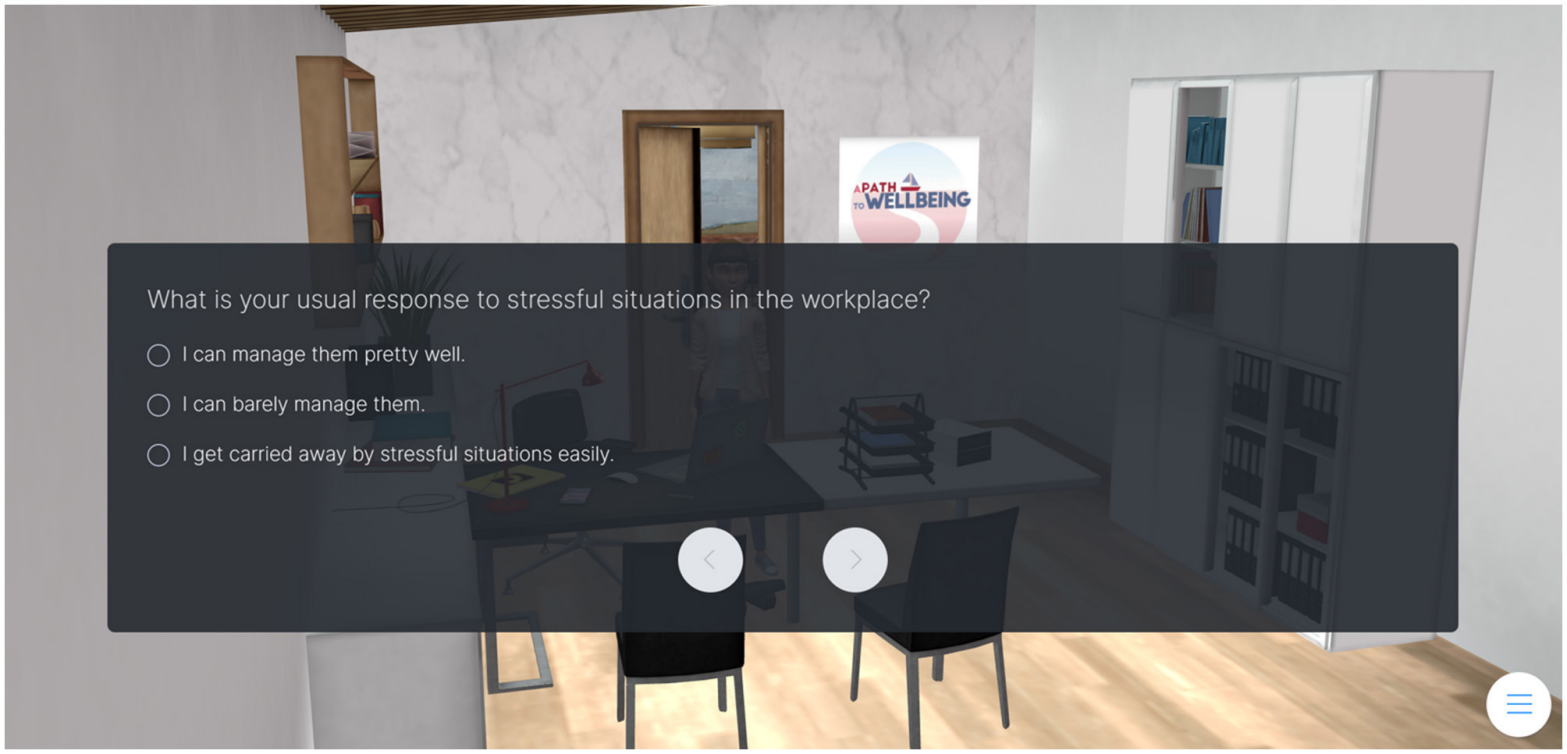
Example of a quiz integrated into the serious game related to own reactions to stressful situations.

Throughout the game, reflection prompts were strategically integrated to encourage teachers to pause and reflect on their experiences, emotions, and thoughts about different aspects of school life related to teachers' wellbeing. These prompts appeared at key decision points as a request to complete a short quiz integrated within the game (e.g., see Figure 4). By prompting reflection, the game created opportunities for teachers to gain deeper insights into their own wellbeing.

#### 3.3.4 Feedback and progress monitoring system

**Figure 5:**
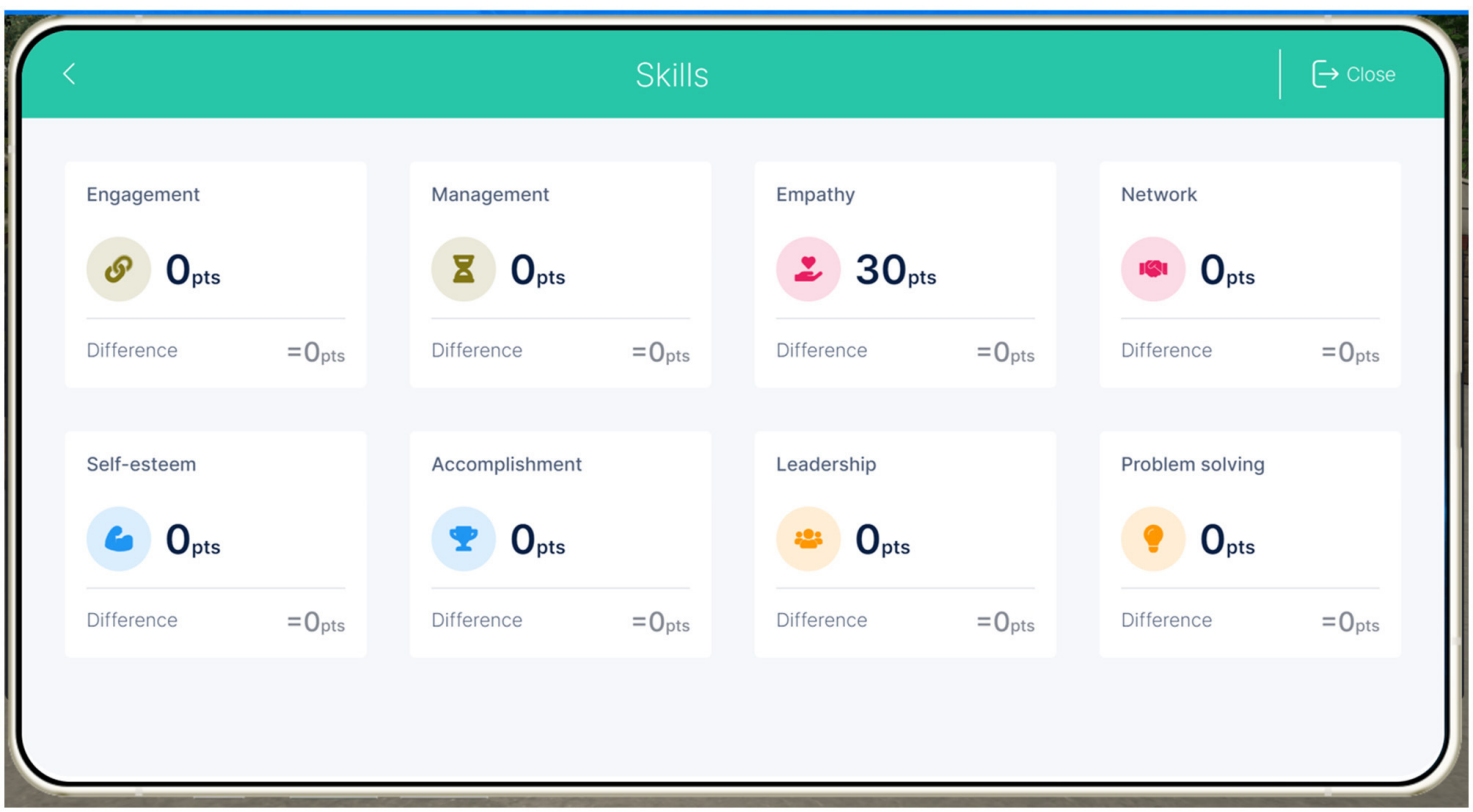
Feedback and progress monitoring system.

The serious game provided visual feedback and progress monitoring features that allowed teachers to track their development and growth over time. This included achieved points and overall performance summaries related to several social and emotional skills (e.g., see Figure 5). By providing feedback, the game fostered self-awareness and encouraged teachers to reflect on their strengths, areas for improvement, and the impact of their actions on their and others' wellbeing.

**Figure 6:**
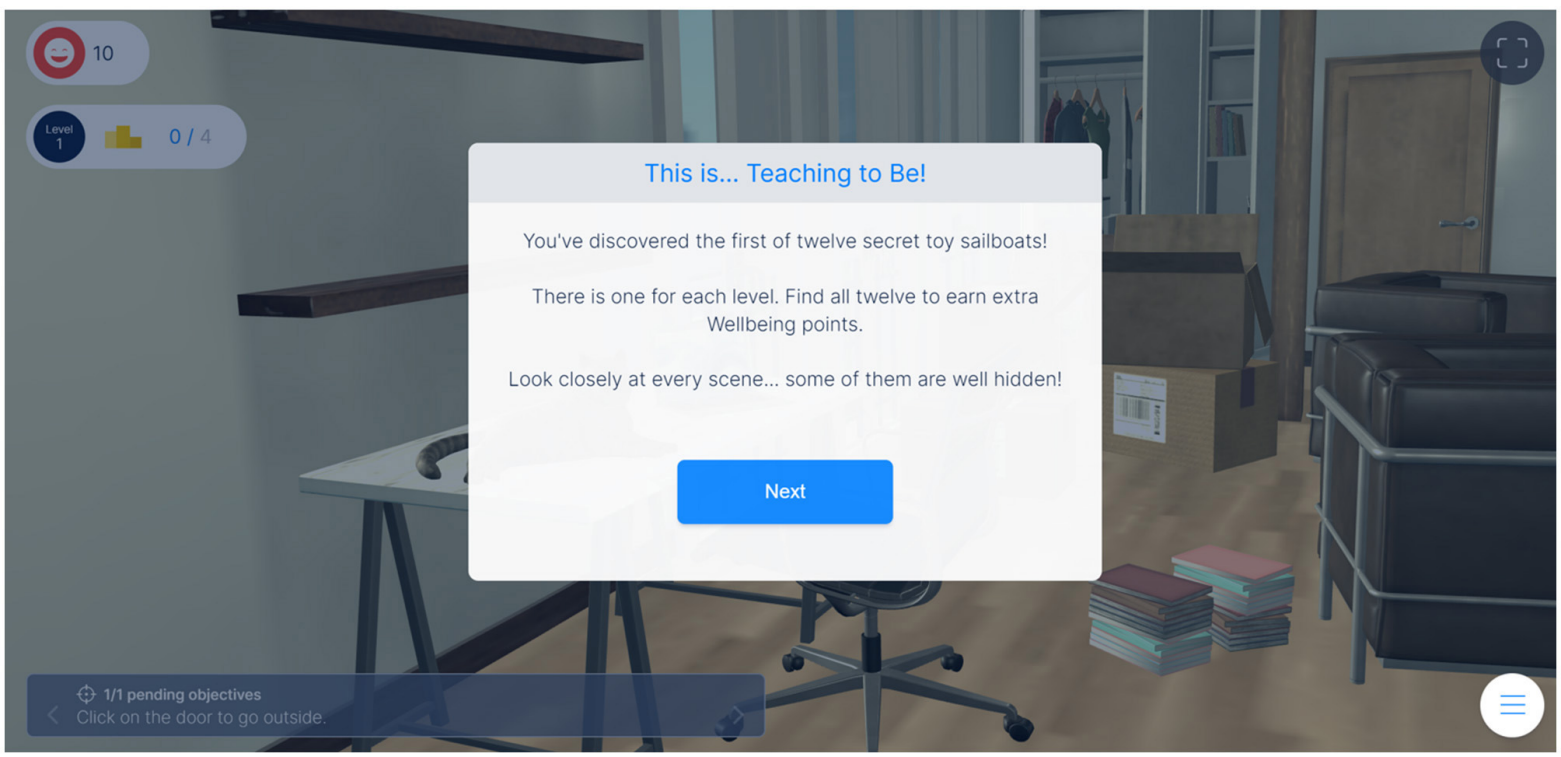
Output of the game after earning extra wellbeing points.

To incentivize continued participation, a system of rewards was incorporated. Participants received virtual points called “extra wellbeing points” for achieving specific milestones (see Figure 6). The incorporation of the reward system provided immediate feedback and reinforcement for teachers' efforts, fostering a sense of accomplishment and satisfaction, and creating a sense of progression and measurable success, allowing teachers to track their growth and development throughout the gameplay experience. The feature of evaluating one's game performance in real-time using such reward mechanisms also facilitates the ability to understand errors and respond immediately and correctly to game requests. Furthermore, the reward system added an element of gamification, making the learning process more enjoyable and engaging (Granic et al., 2014; Faiella and Ricciardi, 2015).

#### 3.3.5 Integrated videos

In order to enhance the learning experience and provide teachers with in-depth knowledge about specific aspects of teachers' wellbeing, the serious game incorporated the integration of short videos. They served as additional resources within the game, offering teachers the opportunity to delve deeper into key topics related to their wellbeing and social and emotional abilities. The short videos were developed for the game, and they covered a wide range of topics pertinent to teachers' wellbeing, such as stress management techniques, self-care strategies, communication strategies and fostering positive relationships. The visual and auditory nature of the videos facilitated the retention and comprehension of key concepts, promoting a more immersive learning experience.

#### 3.3.6 Supplementary handbook

**Figure 7:**
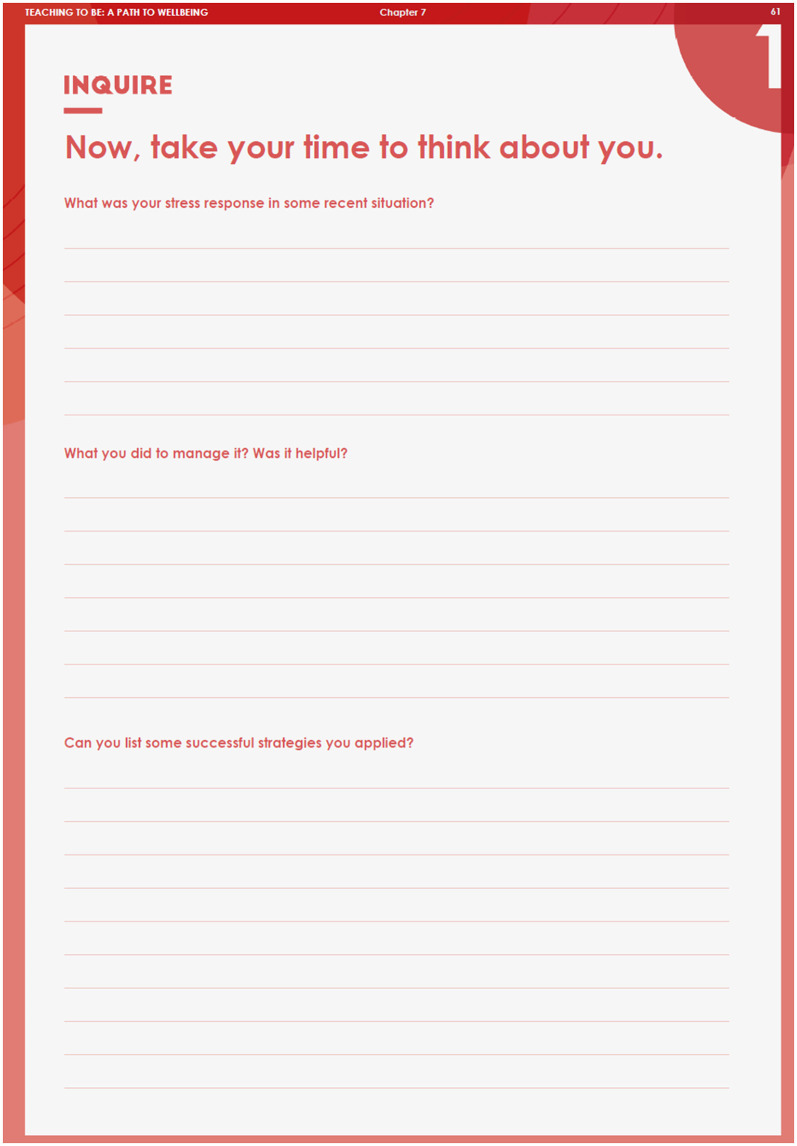
Example reflective activity in the supplementary handbook.

In addition, an accompanying handbook (Talič, 2023) was provided as a supplementary feature to enhance the learning experience and promote self-reflection among the participating teachers. The handbook was distributed in digital format to each participant alongside their access to the serious game. The handbook was designed to be used in parallel with the gameplay, providing a structured framework for reflection and application of the concepts and skills acquired during gameplay. The handbook included sections that corresponded to the various levels and objectives of the serious game. The handbook included several activities ranging from self-assessments and goal-setting exercises to open-ended reflection prompts and journaling opportunities (e.g., Figure 7). These activities were designed to encourage deeper engagement, self-awareness, and application of the concepts and skills acquired in the serious game. It complemented the interactive nature of the serious game with more introspective and reflective activities, fostering a deeper understanding and application of the knowledge and sharing insights with colleagues in offline environments.

## 4 Methods

### 4.1 Participants

The sample comprised 189 teachers from 10 schools located in Lombardy and Piedmont, in northern Italy (mean age = 47.7 years; *SD* = 9.8; min = 19 years; max = 65 years). Table 3 presents the descriptive statistics of the sample. The study was approved by the Ethics Committee of the University of Milano-Bicocca (protocol n. 0129650/21). Participants received details about the goals of the study and the research procedure. Informed consent was obtained for all participants in line with the Declaration of Helsinki. Teachers were free to withdraw from the study at any time, and no monetary or other financial rewards were provided.

**Table 3:** Descriptive statistics of participants (*N* = 189).

**Variables**	**Frequency**	**Percentage**
**Gender**		
Males	18	9.5
Females	170	89.9
Rather not answer	1	0.5
**Education**		
High school graduation	66	34.9
Three-year degree	23	12.2
Master's degree	90	47.6
Postgraduate degree	10	5.3
**Type of school**		
Kindergarten	18	9.5
Primary	105	55.6
I grade secondary school	24	12.7
II grade secondary school	41	21.7
Missing data	1	0.5
**Years of teaching**		
1–5	37	19.6
6–10	23	12.2
11–15	20	10.6
16–20	33	17.5
20–25	33	17.5
21–25	26	13.8
26–30	17	9.0
31–35	19	10.1
Over 36	14	7.4
**Type of contract**		
Full-time	176	93.1
Part-time	8	4.2
Missing data	5	2.5

### 4.2 Procedure

Participants in this study had access to the OWC for 5 months, from the end of November 2022 to the beginning of April 2023. The game was accessible via individual user accounts, which were created and assigned to each participating teacher. Access to the game was provided through Gamelearn (www.game-learn.com), a game-based learning platform allowing teachers to engage with the game at their convenience. Teachers were asked to complete one level per week to permit a steady progression through the game, providing a balance between engagement and the time constraints of teachers' professional responsibilities. In addition, participants were also asked to complete the activities in the handbook which were designed to complement their gameplay experience. Throughout the 5-month duration of the implementation, participants were encouraged to refer to the handbook regularly, particularly after each gameplay session. Both the game and handbook were available in Italian language.

#### 4.2.1 Monitoring strategies to ensure teachers' engagement and progress

The time spent playing the serious game and the number of levels completed by participants were tracked through the game's analytics system. This allowed for ongoing monitoring of participants' engagement and progress throughout the five-month duration of the intervention. To ensure participants' retention and sustained engagement in the OWC, the following approaches were employed. First, the serious game was structured with weekly clear objectives and milestones that provided a sense of progress and accomplishment. Participants were able to track their advancement within the game, fostering a sense of achievement and motivation to continue (Pallavicini, 2020; Zairi et al., 2022). Moreover, regular communication was maintained with participants throughout the study duration to provide updates, reminders, and motivational messages. Notifications were sent via an anonymous internal system integrated into the game to keep participants informed and engaged. These communications also served as gentle reminders of the upcoming activities and the value of their continued participation. These approaches were implemented to foster motivation and a sense of progress, creating a favorable environment for active involvement in the study.

### 4.3 Data collection

The focus group methodology was employed to collect qualitative data and gather in-depth insights from teachers regarding their experiences with the OWC. The focus group sessions were conducted after teachers completed the OWC by two skilled facilitators experienced in qualitative research methodologies. Each group approximately consisted of about 18–20 teachers. The facilitators provided an overview of the purpose and structure of the focus group, ensured a safe and respectful environment, and encouraged active participation from all participants. A total of 10 focus groups were conducted, one for each school whose teachers participated in the OWC, lasting 45 to 60 min. The sessions were audio-recorded with participants' consent to capture the rich and nuanced data. The protocol included one open-ended question (“What changes have you observed in yourself and others after participating in the course?”) aimed to explore the participants' perceptions related to the OWC.

### 4.4 Data analysis procedure

A thematic analysis approach (Braun and Clarke, 2006) was used to examine the results obtained from the focus groups conducted with teachers. The audio recordings of the focus group discussions were transcribed verbatim, ensuring an accurate representation of participants' responses. Two independent researchers were employed for initial coding to capture a broad range of ideas and perspectives expressed by the participants. Meaningful units of data, such as phrases, sentences, or paragraphs, were identified. The initial codes were organized into potential themes and sub-themes based on similarities and differences identified in the data. An iterative process of comparing and contrasting codes was undertaken to identify overarching themes that best represented the essence of the participants' reflections. The themes and sub-themes were discussed, reviewed, and refined among the two researchers to ensure accuracy and coherence and to reach an agreement on their categorization. Final feedback from another researcher was sought to enhance the validity and reliability of the identified themes and sub-themes. The interrater agreement among researchers was *k* = 0.91.

## 5 Results

Thematic analysis revealed six main themes related to the different experiences and perspectives reported by teachers during the focus groups. Table 4 summarizes the identified themes and sub-themes, and provides examples of teachers' statements.

**Table 4:** Focus group: themes, sub-themes, and examples of statements.

**Themes**	**Sub-themes**	**Examples of statements**
1. Emotional competence	1.1 Self-awareness	“The game made me more attuned to my own feelings and reactions. I learned to think about how I feel, to be able to better recognize negative emotions and maintain a positive mindset”
	1.2 Emotional regulation	“I often struggled with managing my emotions in the face of daily challenging situations. However, through the various suggestions, including reflective discussions with my colleagues, I have developed a greater ability to regulate and understand my emotions”
	1.3 Stress and burnout management	“I gained a deeper awareness of how negative emotional states can influence my behaviors. This allowed me to recognize and acknowledge my emotions enabling me to respond more effectively to stressful situations and developing a greater sense of calmness. Mindfulness exercises, such as deep breathing and body scans, have become valuable tools in helping me stay grounded even during high-stress situations”
2. Self-caring	2.1 Self-compassion	“Before the OWC, I often found myself being overly self-critical and judgmental, constantly striving for perfection and feeling inadequate. Now I try to be less critical of myself, to allow myself to make mistakes and not feel guilty. It encouraged me to reflect on my teaching practices, challenges, and successes accepting my limitations, mistakes, and areas for growth with compassion and understanding”
	2.2 Pay attention to one's own mental health	“Engaging with the game has been instrumental in helping me prioritize and pay attention to my own mental health. Prior, I often neglected my wellbeing, putting the needs of my students and overall school demands above my own. Now I feel I have learned the importance of self-care. The game served as a wake-up call, reminding me of the necessity to nurture my own wellbeing”
3. Social awareness	3.1 Empathy toward others	“I have noticed that I have developed more attention toward the feelings of my colleagues. I tried to better observe their behaviors to understand how they felt to be more empathetic”
	3.2 Valuing the strengths of others	“Participating in the game has had a profound impact on how I perceive and value others' abilities. Prior, I often focused on my own strengths and accomplishments. I discovered that some of my colleagues had skills that I didn't know, and I learned to value them more”
	3.3 Caring for others	“I have developed a deeper attention to the emotional state of my colleagues and a more intentional attitude to care for others. I realized I am now more prone to understand the diverse needs and perspectives of those around me. I am more mindful of the impact of my words, gestures, and choices on others. Small acts of care, such as providing words of encouragement, or offering a helping hand, became an integral part of my daily practice”
4. Relationship skills	4.1 Communication	“The game encouraged me to practice active listening and choose my words carefully. It improved my communication skills, allowing me to connect better with my peers and students and create a more open and non-judgmental dialogue”
	4.2 Collaboration	“The game created a community of teachers where we could exchange ideas and support one another. I realized that we are all striving toward a common goal of creating a nurturing and enriching learning environment for our students”
	4.3. Leadership	“I often doubted my abilities and hesitated to take on leadership roles. However, through the game's scenarios, decision-making challenges, and collaborative discussions with my colleagues, I have gained more confidence in my leadership skills. I now feel empowered to share my ideas, take initiative, and advocate for positive change within my school community”
5. Decision-making	5.1 Critical thinking	“The game encouraged me to question my assumptions and beliefs, especially in approaching challenging social problems. It pushed me to better analyze information and look for alternative solutions to reach greater outcomes for me, my peers, and students”
	5.2 Evaluating the consequences of own one's actions	“The game helped me to think critically and consider the potential impact of my decisions. This improved my ability to make effective and responsible ethical choices in my teaching practice”
6. School climate	6.1 Cohesion	“Sharing our experiences and hearing the stories of others, starting from the scenarios suggested in the game, helped me develop a deep sense of connection toward my peers. We engaged in meaningful conversations starting from the challenges presented in the game. We shared teaching practices, supported each other through difficulties, and celebrated our achievements and this led to a strong sense of cohesion”
	6.2 School belonging	“I often felt isolated and disconnected within the school community, overwhelmed by the demands of my profession. However, through the game suggestions and reflections shared also with my colleagues, I began to realize that I am not alone in facing challenges and that we are all on this journey together. I no longer feel isolated but rather an integral part of our school community”

### 5.1 Theme 1: emotional competence

The first prominent theme that emerged from the focus group analysis was the participants' reported improvements in their emotional competence.

*Subtheme 1.1: Self-awareness*. Participants revealed that the course helped them to better recognize, and understand their own emotions as well as those of their colleagues and students. Engaging with the serious game provided teachers with opportunities to explore and navigate various challenging scenarios, allowing them to develop a deeper understanding of emotions and their impact on teaching and learning.

*Subtheme 1.2: Emotional regulation*. Teachers described increased awareness of their own emotional triggers enabling them to better manage their responses both inside and outside the classroom. By engaging with the game's activities and reflecting on emotional experiences within the virtual environment, participants gained insights into their emotional responses and learned strategies for regulating their emotions effectively. This increased ability enabled teachers to approach their classrooms with a greater sense of self-efficacy and self-esteem.

*Subtheme 1.3: Stress and burnout management*. Participants reported that the OWC gave them the possibility to vary and improve their coping strategies and provided some stress reduction techniques such as mindfulness exercises and deep breathing techniques. They reported being more attuned to their emotional, behavioral, and physical states to take proactive steps to prevent significant stress before reaching a point of exhaustion and burnout. The serious game also yielded a sense of playfulness, relaxation, and escape from work-related stressors. Hence, the OWC provided a temporary respite from the demands of their role, enabling them to recharge and approach their work with greater clarity and enthusiasm.

### 5.2 Theme 2: self-caring strategies

Another significant theme that emerged from the analysis was the participants' adoption of enhanced self-care practices to nurture their own wellbeing. The serious game intervention acted as a catalyst for self-reflection and personal growth, empowering teachers to implement self-care practices effectively.

*Subtheme 2.1: Self-compassion*. Teachers described an increased ability to show kindness, understanding, and acceptance toward themselves, particularly in the face of challenges and setbacks. In other words, engaging with the serious game provided teachers with opportunities to develop a more compassionate attitude toward themselves and challenge self-critical and self-judgmental thoughts. Participants reported that engaging with the game's activities and narratives helped them reframe negative self-talk and develop a more supportive and compassionate inner dialogue. This shift in mindset allowed teachers to approach their own challenges and weaknesses with greater kindness and understanding, leading to improved self-esteem and overall wellbeing.

*Subtheme 2.2: Pay attention to one's own mental health*. Participants described enhanced awareness and prioritization of their own wellbeing and the adoption of effective self-care practices. Engaging with serious game activities and the handbook suggestions provided teachers with a space for self-reflection about their wellbeing. Teachers expressed that the course offered valuable resources and strategies for promoting work-life balance and maintaining overall wellbeing. Participants shared how they implemented these strategies, such as setting boundaries and effective time management resulting in improved self-care habits and a more sustainable approach to their profession. Moreover, the OWC fostered a supportive community among teachers, encouraging them to share self-care tips, experiences, and mutual encouragement. The OWC encouraged the adoption of proactive approaches to address and prevent mental health difficulties within the school. Teachers shared how the game prompted discussions and awareness about their psychological wellbeing among the entire school community. By normalizing conversations about self-care strategies, the serious game created a supportive environment that prioritized the holistic wellbeing of both teachers and students.

### 5.3 Theme 3: social awareness

Teachers reported an enhanced ability to show empathy, appreciation, and caring behaviors toward others.

*Subtheme 3.1: Empathy toward others*. Teachers expressed that the serious game prompted them to reflect on their own biases and assumptions and this, in turn, increased their capacity to empathize with others' emotions. Participants reported that engaging with the game's diverse characters and storylines exposed them to a range of emotional experiences. This exposure fostered empathy and a deeper understanding of the emotions of peers and their students.

*Subtheme 3.2: Valuing the strengths of others*. Participants gained insights into the experiences of individuals from diverse perspectives and developed a deeper appreciation for the diverse strengths and needs of their students and colleagues. Teachers shared how this newfound capacity positively influenced their interactions with students, allowing them to provide more personalized support.

*Subtheme 3.3: Caring for others*. The OWC provided teachers with opportunities to practice navigating challenging discussions and develop strategies for creating safe spaces where students could express their thoughts and experiences openly. As a result, teachers felt more equipped to facilitate conversations about students' emotional and social difficulties, which translated into more supportive and inclusive school environments where care is prioritized.

### 5.4 Theme 4: relationship skills

The OWC positively affected teachers' relationship skills related to the ability to communicate and listen actively, establish collaborative relationships, seek, and offer support when needed, and apply effective leadership skills.

*Subtheme 4.1: Communication*. Participants described improvements in their ability to effectively communicate with colleagues, students, and families. They emphasized how the OWC simulated dialogues and communication challenges and provided them with practical strategies for expressing their thoughts, actively listening, and resolving conflicts. Teachers reported feeling more confident in delivering constructive feedback to students and engaging in collaborative discussions with their peers.

*Subtheme 4.2: Collaboration*. Teachers highlighted the value of peer support and collaborative learning within the serious game and applying theoretical and practical knowledge they gained also from the handbook's activities. Therefore, participants reported an increased recognition of the valuable role that peer support plays in their professional development and overall wellbeing. Engaging with the serious game provided teachers with opportunities to connect with their peers, share experiences, and seek and provide advice and encouragement. Through the game's shared reflections, teachers felt more comfortable reaching out to their colleagues for assistance and sharing their own knowledge and expertise in real-life settings. The OWC contents provided participants with the opportunity to engage in meaningful conversations, exchange ideas, and provide mutual support also in the off-line school contexts. This experience reduced feelings of isolation and contributed to teachers' overall professional growth. Furthermore, teachers described how their enhanced collaboration and teamwork positively affected students' engagement, motivation, and academic achievement, ultimately benefiting the entire educational community.

*Sub-theme 4.3: Leadership*. Participants reported an enhanced understanding of leadership principles and an increased ability to apply those principles in their teaching practice. Teachers highlighted the positive effect of the OWC on their ability to inspire and motivate their students. By engaging with the game's motivational aspects, such as setting goals, providing feedback, recognizing and valuing strengths, teachers gained insights into techniques for fostering students' engagement and intrinsic motivation. Consequently, participants felt better equipped to lead and influence school members positively and productively and more empowered to take on leadership roles within their classes and schools and make meaningful contributions to the improvement of their teaching practice and the entire school community.

### 5.5 Theme 5: decision-making

Teachers shared that engaging with the OWC enhanced their critical thinking skills, problem-solving abilities, and overall decision-making capacity.

*Sub-theme 5.1: Critical thinking*. The interactive nature of the game, which presented teachers with various scenarios and challenges, allowed them to practice making informed decisions in a risk-free environment. Teachers described how the serious game intervention provided them with opportunities to explore different perspectives, evaluate the consequences of their decisions, and reflect on the outcomes. By engaging in simulated decision-making experiences, participants developed a greater sense of confidence and competence in making informed choices in real-life teaching situations. Teachers also emphasized that the serious game intervention expanded their repertoire of strategies, enabling them to make more evidence-based decisions. The game incorporated research-backed psychological techniques and presented teachers with evidence-based information to support their decision-making. Teachers described how the game prompted them to reflect on their own teaching practices, consider alternative approaches, and identify areas for improvement in personal and professional development.

*Sub-theme 5.2: Evaluating the consequences of own actions*. The process of reflection and evaluation allowed teachers to develop a more thoughtful and deliberate approach to their teaching practice, considering the short-term and long-term effects of their actions on students' academic, social, and emotional development. Hence, teachers reported an increased awareness of the potential impact of their professional decisions and behaviors on their students, colleagues, and the overall learning environment. Teachers expressed that the OWC helped them develop a greater sense of responsibility and accountability for their actions as educators.

### 5.6 Theme 6: school climate

Thematic analysis revealed that the OWC had a positive influence on teachers' overall perception of the school climate. Teachers expressed a shared commitment to creating a positive school climate and recognized the important role they played in shaping the overall atmosphere.

*Subtheme 6.1: Cohesion*. Teachers described that their participation in the game fostered a greater sense of unity among staff members, leading to enhanced school cohesion. The shared experience of engaging in the OWC created a common language and understanding among teachers, promoting a stronger sense of belonging within the school community. The positive impact of the OWC on school cohesion extended beyond the interactions among teachers. Participants noted that the improved cohesion among staff members influenced their interactions with students, parents, and the broader school community.

*Subtheme 6.2: School belonging*. Teachers reported that their participation in the OWC positively affected the atmosphere and dynamics within their schools. Teachers expressed that their increased wellbeing positively influenced their interactions with colleagues and administrative staff enhancing the sense of belonging to the school.

## 6 Discussion

This study explored the teachers' experiences related to the OWC. The combined use of the serious game and the handbook fostered a more holistic and multifaceted experience, supporting participants in transferring their learning into real-world practice and promoting a deeper understanding of the concepts and skills addressed in the online environment. By incorporating the handbook as a parallel resource to the serious game, this methodology aimed to provide a comprehensive and integrated approach to the participants' learning and reflection process.

The findings gathered via the analysis of focus group data provided valuable insights into the potential of serious games as a tool for promoting professional wellbeing among teachers. The thematic analysis revealed several key themes and sub-themes that emerged from the focus group discussions, highlighting the positive impact of the serious game on various aspects of teachers' wellbeing which are discussed below in further detail.

First, the serious game intervention was found to promote teachers' emotional competence. The research has largely recognized that teaching is a demanding and emotionally challenging profession (e.g., Cavioni et al., 2023b). Teachers who are more attuned to their own emotions are better equipped to cope with stress, regulate their emotional responses, and prevent burnout (De Stasio et al., 2019). This will, in turn, foster a positive and inclusive learning environment as teachers can respond to students' emotional needs more effectively (Jennings and Greenberg, 2009; Braun et al., 2020).

Second, the OWC has been successful in boosting self-care routines among teachers. Participants reported an increased awareness of the importance of self-care and the adoption of various self-care strategies, such as mindfulness exercises and relaxation techniques. By incorporating elements of self-care into gameplay mechanics, the serious game promotes the cultivation of self-care habits. It also provided a virtual space for self-reflection and prompted participants to prioritize their own wellbeing and encouraged teachers to share their insights among peers in the offline school environments. By prioritizing their wellbeing, teachers not only better navigate the daily challenges and demands of their profession but also experience a reduction in burnout. This holistic approach to self-care enhances their resilience enabling more effective classroom management (Schonert-Reichl et al., 2017; Miller and Flint-Stipp, 2019). Consecutively, this allows them to be more present, engaged, and effective in their interactions with students, creating a conducive learning environment (Walter and Fox, 2021; Cavioni et al., 2023b).

The findings also revealed that the serious game positively influenced teachers' social awareness, including empathy, valuing others, and showing caring behaviors. Hence, participants reported an enhanced ability to understand and connect with the emotions and experiences of their students and colleagues. The research has shown that when teachers demonstrate understanding and compassion toward their students, they build strong trust and a sense of connection that contribute to creating nurturing educational spaces where students feel comfortable expressing their thoughts, concerns, and emotions (Collie et al., 2016). Additionally, students who perceive their teachers as empathetic are more likely to engage actively in learning, seek guidance when needed, and develop a positive attitude toward school demands (Hamre and Pianta, 2001; Serbati et al., 2020).

Participants reported increased collaboration, and teamwork, enhanced confidence in their leadership abilities, and a willingness to take on new challenges and responsibilities. Such skills are essential for the promotion of a positive school culture. When teachers demonstrate strong collaboration skills, they can effectively collaborate with their peers, share resources and expertise, and engage in meaningful professional dialogue which ultimately improves their work engagement and wellbeing (Klassen et al., 2012). Moreover, establishing supportive connections with both students and colleagues was found to have a positive impact on job satisfaction. This collaborative approach fosters a supportive work environment, leading to enhanced instructional practices, positive teacher-student relationships and increased student achievement (Strati et al., 2017; Ortan et al., 2021).

Our findings suggested that serious games could positively affect teachers' decision-making abilities by enhancing critical thinking and promoting reflective practices. By offering a safe and supportive space for practicing decision-making, our serious game empowered teachers to make more informed choices that ultimately benefit their students and teaching practice. This also highlights the potential of such serious games as a tool for professional development in ethical decision-making. The development of these abilities is of paramount importance as teachers hold a position of influence and responsibility, shaping the lives of their students and playing a crucial role in their moral and ethical development. Ethical decision-making ensures that teachers spotlight the wellbeing of their students (Woo et al., 2022). By developing the ability to make ethical decisions, teachers can critically analyse challenging school situations, consider multiple perspectives, and choose actions that align with fairness and justice principles.

Lastly, these positive changes reported by teachers appeared to produce a ripple influence on the school climate (Cavioni et al., 2023c; Martinsone and Žydžiunaite, 2023). The serious game intervention was found to produce positive changes in the school climate in terms of cohesion and a sense of belonging. A positive school climate improves teachers' overall wellbeing and job satisfaction (Murray and Greenberg, 2000). Teachers who work in a positive and supportive school climate experience higher levels of job satisfaction, lower levels of stress and burnout, and increased professional fulfillment (Lester et al., 2020; Cavioni et al., 2023b; Conte et al., 2024). In addition, teachers fostering a positive school climate serve as models for respectful school communities (McLean et al., 2017). Students who experience a positive school climate are more likely to display prosocial behaviors, empathy, and engagement, as well as develop a sense of civic responsibility and show higher academic success (Cefai and Cavioni, 2014; Cefai et al., 2021).

## 7 Conclusion

The positive outcomes observed in teachers' emotional and social competence, self-care strategies, decision-making, and school climate suggest that serious games can be an innovative tool for supporting the holistic development of teachers. The findings of this study represent a significant breakthrough in the field of teachers' wellbeing, particularly considering the unprecedented challenges posed by the COVID-19 pandemic (Cowie and Myers, 2020). Previous research has largely overlooked the potential of serious games to support and promote the wellbeing of educators, especially in the context of such a global crisis. To our knowledge, this is the first study that filled an important gap in the literature by exploring the potential of a serious game specifically designed to address the unique wellbeing needs of teachers, considering the profound impact of the COVID-19 pandemic (Lee and Yin, 2021; Collie, 2022). The incorporation of the serious game intervention as a tool for coping skills-building and self-care demonstrates the study's responsiveness to the evolving needs of teachers in the face of unprecedented circumstances.

This study exhibits several innovative aspects and strengths, which contribute to its significance and potential impact in the field of teachers' wellbeing. Firstly, the integration of evidence-based research and participatory action research analysis in building the contents of OWC has ensured that the serious game was aligned with the realities and experiences of teachers. This innovative approach acknowledges the importance of providing tailored interventions that meet the specific demands of the teaching profession and support the overall wellbeing of teachers. Secondly, the incorporation of various features within the serious game, such as the interactive elements, reward system, inventory objects, and multimedia resources, showcases the innovative nature of this study. By combining these elements, the serious game provides an engaging, immersive, and multifaceted learning experience for teachers, fostering their growth, skill development, and self-reflection (Bakhanova et al., 2020). Furthermore, the integration of a handbook and the encouragement of offline discussions among colleagues further enhance the potential impact of the serious game beyond individual gameplay sessions. The combined use of the serious game and the handbook offered a well-rounded and multifaceted learning experience, promoting a deeper understanding of the content and encouraging practical application in teachers' daily professional lives.

The study outcomes also have implications for educational policymakers aligning with broader educational policies that prioritize teacher and student wellbeing (World Health Organization, 2021). The results suggested that integrating serious games into teachers' professional development initiatives can be a valuable investment for enhancing educators' wellbeing. The findings of this study could motivate policymakers to place a greater emphasis on teacher wellbeing by endorsing programs that bolster teacher support, mitigate stress and burnout risks, and consequently elevate both classroom dynamics and student achievements. This perspective advocates for a holistic approach in educational policy, where the wellbeing of educators is seen as foundational to creating optimal learning environments and enhancing educational outcomes (Brackett et al., 2012; Schonert-Reichl et al., 2017).

Our study primarily utilized qualitative research methodologies, particularly focus groups, to delve into the experiences and perceptions of teachers who engaged with the course. This qualitative approach yielded rich, detailed insights into the participants' views and interactions with the course. However, it's crucial to recognize some limitations of this methodology. Firstly, the reliance on qualitative data from focus groups means that the study lacks quantitative measures, which are essential for providing statistical evidence of the OWC's impact. The inclusion of quantitative evaluations, such as pre-and post-intervention assessments, as well as the presence of a control group, would enable a more objective measurement of any changes in wellbeing outcomes attributable to the course (Dugard and Todman, 1995). The absence of a control group in our study makes it challenging to definitively attribute any improvements in wellbeing outcomes solely to the OWC, as these changes could potentially be influenced by other external factors (Kamper, 2018). Secondly, our study sample might not adequately represent the diverse range of teachers with varying backgrounds and contexts, potentially limiting the generalizability of the findings to a larger population of teachers. The results may be more applicable to the specific context and group of participants involved, and caution should be exercised when extrapolating the findings to other teacher populations. Thirdly, the focus groups applied for data collection may not have captured the full range of teachers' perspectives and experiences. This limitation stems from the observation that, despite all teachers' presence in the focus groups, the extent of participation varied significantly among them. Certain teachers were more vocal, sharing their views and experiences extensively, while others remained more reserved, contributing less to the discussions. This dynamic could lead to a form of selection bias, where the data gathered disproportionately reflects the viewpoints of the more outspoken participants, potentially overlooking the quieter, yet equally valuable, insights of less vocal teachers. Fourthly, participants in the focus groups may have felt compelled to provide socially desirable responses or conform to perceived expectations. Moreover, self-report data can be influenced by participants' memory, biases, or subjective interpretations, potentially introducing further limitations to the reliability and accuracy of the collected information (Iaconelli and Wolters, 2020). Consequentially, the reliance on results may include bias and potential inaccuracies which can affect the reliability and validity of the data. Additionally, qualitative data analysis involves interpretation, and the findings may be subject to the researcher's subjective perspectives and biases. The researchers' preconceived notions or personal beliefs may inadvertently influence the coding, analysis, and interpretation of the data, potentially introducing researcher bias. Lastly, this study focused on short-term outcomes, such as immediate changes in wellbeing outcomes based on teachers' self-reported perceptions. Acknowledging these limitations demonstrates a thorough understanding of the study's potential weaknesses and opens avenues for future research to address these limitations.

In terms of future research, longitudinal studies that follow participants over an extended period would provide a more comprehensive understanding of the intervention's long-term impact exploring other key aspects of teachers' wellbeing such as job satisfaction and the overall quality of life. Additionally, examining the perspectives of students about the observed changes in teachers would provide a more comprehensive understanding of the game's impact. Further development in this field can build upon the study findings to explore new game mechanics and interactive features that continue to support the wellbeing of teachers and enhance their professional experiences.

To conclude, the results emphasize the potential of serious games as an effective tool for promoting wellbeing among teachers. By investigating the positive outcomes of the OWC serious game intervention, this study has paved new pathways for improving teacher wellbeing and nurturing their social-emotional growth by connecting technology with wellbeing support for educators.

## Data availability statement

The raw data supporting the conclusions of this article will be made available by the authors, without undue reservation.

## Ethics statement

The study was approved by the Ethics Committee of the University of Milano-Bicocca and conducted in accordance with the local legislation and institutional requirements. The participants provided their written informed consent to participate in this study.

## Author contributions

VC: Conceptualization, Data curation, Formal analysis, Methodology, Writing—original draft, Writing—review & editing. EC: Conceptualization, Data curation, Formal analysis, Writing—review & editing. VO: Conceptualization, Funding acquisition, Investigation, Project administration, Supervision, Writing—review & editing.
